# Examining childhood experiences and personality functioning as potential predictors for the speed of recovery during psychotherapy of patients with anxiety disorders

**DOI:** 10.3389/fpsyt.2024.1381105

**Published:** 2024-05-09

**Authors:** Jonathan Nowak, Christoph Nikendei, Ivo Rollmann, Maximilian Orth, Hans-Christoph Friederich, David Kindermann

**Affiliations:** ^1^ Department of General Internal Medicine and Psychosomatics, University Hospital Heidelberg, Heidelberg, Germany; ^2^ DZPG (German Centre for Mental Health – Partner Site Heidelberg/Mannheim/Ulm), Heidelberg, Germany

**Keywords:** adverse childhood experiences, protective childhood experiences, anxiety disorders, personality functioning, psychotherapy, recovery

## Abstract

**Background:**

Adverse childhood experiences were previously identified as relevant risk factors for the development of anxiety disorders. Furthermore, anxiety disorders were shown to be associated with impairments of personality functioning. The objective of this study was to investigate adverse and protective childhood experiences as well as personality functioning, as defined by the Operationalized Psychodynamic Diagnosis system, as potential predictors for the speed of recovery during psychotherapy for patients with anxiety disorders.

**Methods:**

The sample consisted of *n* = 312 completed psychotherapies. The speed of recovery, defined as symptom abatement over time, was calculated using a two-stage hierarchical linear model. The effects of adverse and protective childhood experiences as well as personality functioning on the speed of recovery during psychotherapy were then examined using a structural equation model.

**Results:**

The presence of adverse childhood experiences predicted a lower speed of recovery during psychotherapy. In addition, a higher number of adverse childhood experiences was associated with greater impairments in the abilities of perception and regulation as dimensions of personality functioning. A higher number of protective childhood experiences was associated with fewer impairments in the communication and attachment dimensions. Impairments in personality functioning in patients with anxiety disorders did not predict the speed of recovery during psychotherapy.

**Conclusions:**

Among patients with anxiety disorders, adverse childhood experiences lead to a lower speed of recovery during psychotherapy. Therefore, childhood adversity should be routinely assessed before and thoroughly addressed during psychotherapy in patients with anxiety disorders.

## 1 Introduction

Anxiety disorders are among the most common mental disorders in the general population worldwide, with an estimated 12-month prevalence of approximately 11.6% (1). Previous studies have consistently found strong associations between anxiety disorders and adverse childhood experiences (ACEs). ACEs are defined as „abuse and household dysfunction during childhood” (2) and include emotional, physical and sexual abuse, physical and emotional neglect, household exposure to substance abuse, mental illness, domestic violence, parental separation or divorce, and criminal behavior (3). In a recent epidemiological study, 30% of anxiety disorder cases in North America and more than 25% of anxiety disorder cases in Europe were attributable to ACEs (4). Risk associations for the development of anxiety disorders have consistently been found for various types of adverse and traumatic childhood experiences, including sexual abuse, physical abuse, emotional abuse, and neglect (5–9). Furthermore, ACEs were previously identified as being associated with greater clinical complexity in patients with psychiatric disorders, e.g. more comorbidities, higher than typical intensity or duration of interventions, and poorer outcomes of treatment (10–12). Recent research has also begun investigating the role of protective childhood experiences (PCEs) and their interaction with ACEs (13–15). Findings regarding PCEs in anxiety disorders include moderating effects of PCEs on ACEs that influence the development of anxiety symptoms during adolescence (16) and are associated with a lower risk for later adolescent anxiety (17).

In addition to their connection with adverse and protective childhood experiences, anxiety symptoms and disorders have previously been found to be closely associated with personality traits, such as high neuroticism, low extraversion, and personality disorders (18). In particular, Cluster C personality disorders, namely avoidant personality disorder, dependent personality disorder, and obsessive-compulsive personality disorder, were shown to be associated with anxiety symptoms (18, 19). More recent studies examining the relationship between personality characteristics and anxiety symptoms focused on the concept of personality functioning in terms of the dimensional, alternative model for personality disorders according to DSM-5 (e.g. 20). With respect to this, Doering et al. (2018) demonstrated that anxiety disorders are associated with significant impairments of personality functioning, which were, in turn, shown to be significantly increased by comorbid personality disorders (21). Another prominent approach of assessing personality functioning on a dimensional level is the Operationalized Psychodynamic Diagnosis System, Version 2 (OPD-2), which is a multiaxial diagnostic and classification system developed on the basis of psychodynamic concepts (22–25). According to the OPD-system, personality functioning is operationalized as consisting of four dimensions, namely the basic mental abilities of perception/cognition, regulation, communication, and attachment (24, 25). In a recent cross-sectional study examining personality functioning, according to the OPD-system, in different anxiety disorders, it was illustrated that anxiety disorders differ with regard to impairments of specific dimensions of personality functioning (26). Furthermore, longitudinal studies in patients with anxiety and mood disorders also indicated changes in personality functioning during the course of psychotherapy (27–29). It is assumed that improvement in personality functioning may be associated with improvement of symptoms during psychotherapy (27, 30). However, it remains unclear how impairments in personality functioning at the onset of psychotherapy may predict treatment outcome in anxiety disorders.

Aside from the findings that both ACEs and personality functioning are each associated with treatment outcome in psychotherapy, ACEs have repeatedly been linked to personality functioning (23, 31–35). For example, it was found that the interaction between ACE and personality functioning predicts psychopathology, including anxiety symptoms (36). Assessing a heterogeneous sample of psychotherapy patients, Kindermann et al. (2023) recently showed that ACEs were directly associated with a lower speed of recovery during psychotherapy. Furthermore, ACEs were demonstrated to have indirect effects on the speed of recovery by being associated with impairments in the communication dimension of personality functioning, which, in turn, was associated with a slower improvement of symptoms (37).

As far as we are aware, no specific studies have been conducted analyzing possible predictive effects of childhood experiences on the speed of recovery during psychotherapy of patients with anxiety disorders. Furthermore, with respect to the previous study by Kindermann et al. (37), it remains unclear whether impairments in specific dimensions of personality functioning might also be predictive of the speed of recovery in patients with anxiety disorders. With this in mind, evaluating ACEs and impairments in specific dimensions of personality functioning before starting psychotherapy could be a relevant component of treatment planning. Based on previous studies, our hypotheses were the following: (a) Adverse and protective childhood experiences in patients with anxiety disorders predict the speed of recovery during psychotherapy, defined as symptom abatement (according to Hopkins Symptom Checklist, SCL-K11) over time; (b) Adverse and protective childhood experiences show differential associations with individual dimensions of personality functioning in patients with anxiety disorders; (c) Impairments in individual dimensions of personality functioning predict the speed of recovery during psychotherapy in patients with anxiety disorders.

## 2 Materials and methods

### 2.1 Study design

This study is a retrospective study of routinely assessed longitudinal data during outpatient psychodynamic psychotherapy within the Heidelberg Institute of Psychotherapy, Heidelberg, Germany.

### 2.2 Participants

#### 2.2.1 Patients

The present study is based on the data of a routine survey of psychotherapy sessions at the Heidelberg Institute of Psychotherapy, Heidelberg, Germany, in the period between January 2013 and July 2021 (38). At the end of the survey period, some treatments had already been completed, as far as documented in the institute, while others were still ongoing. This study is based on N_Completed_ = 648 completed psychotherapies from N_Total_ = 1646 psychotherapies that took place between January 2013 and July 2021. Among all completed psychotherapeutic treatments, N_Anxiety_ = 312 psychotherapies were treatments of patients who had been diagnosed with at least one anxiety disorder. Patients were included after providing written informed consent.

#### 2.2.2 Therapists

Therapists underwent a postgraduate training program in psychodynamic therapy at the Heidelberg Institute of Psychotherapy. N_Therapist_ = 172 therapists participated in this study. Therapists had at least 1.5 years of clinical experience and either a degree in psychology (M.Sc. or Ph.D.) or were medical residents (MD). Each therapist treated M = 9.4 patients (SD = 5.4). Every fourth session was supervised by an experienced psychodynamic psychotherapist with at least 5 years of clinical experience.

#### 2.2.3 Inclusion criteria

The present study applied both patient-related and therapist-related criteria for a psychotherapeutic treatment to be included: Patients (I) had to be proficient in German or English, (II) must have had at least one diagnostic session with their psychotherapist, and (III) had to have been diagnosed with at least one anxiety disorder (i.e. agoraphobia, social phobia, specific phobia, panic disorder, generalized anxiety disorder). Furthermore, therapists (IV) had to indicate that psychotherapy had been completed. We based the study on completed psychotherapeutic treatments, as it is unclear to what extent a patient’s symptoms can still improve while psychotherapy has not yet concluded.

### 2.3 Ethics approval and consent to participate

The study protocol was developed according to the Helsinki II declaration (39). Prior to recruitment of patients and therapists, the study was approved by the independent Ethics Committee of the Medical Faculty of the University of Heidelberg (S-195/2014). Written informed consent was obtained from all study participants.

### 2.4 Procedure

#### 2.4.1 Diagnostic assessment

**Figure 1:**
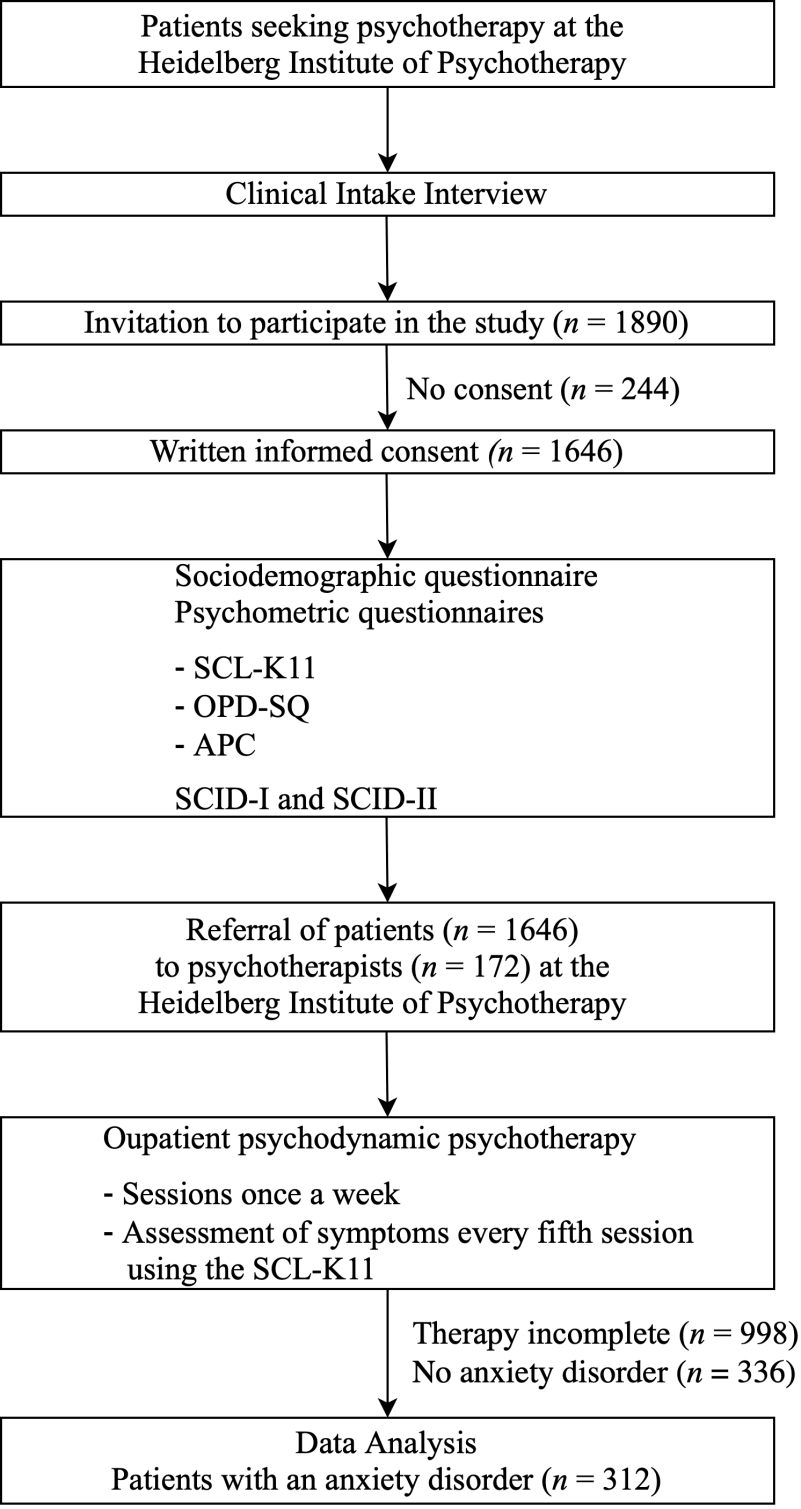
Procedure. The present study was based on data collected between January 2013 and July 2021. At the end of the survey period, some treatments had already been completed, while others were still ongoing. Data analysis was based on completed psychotherapeutic treatments. SCL-K11 = Short Version of the Hopkins Symptom Checklist; OPD-SQ = Operationalised Psychodynamic Diagnosis Structure Questionnaire; APC = Questionnaire for the Assessment of Adverse and Protective Childhood Experiences; SCID = Structured Clinical Interview.

All patients seeking psychotherapy underwent the following procedure (see **Figure 1**): First, a clinical intake interview was conducted to assess indication for psychodynamic psychotherapy (38). During the interview, patients were invited to participate in the study. They were then informed about the study and asked for written informed consent. After this, patients had to answer the sociodemographic and psychometric questionnaires and a standardized diagnostic interview (SCID-I and SCID-II: 40, German version: 41) took place with a trained graduate student (at least B.Sc. in psychology). Patients were then referred to a therapist of the Heidelberg Institute of Psychotherapy involved in the study.

#### 2.4.2 Psychotherapy

Treatment was conducted in the form of outpatient psychodynamic psychotherapy. Psychotherapy sessions took place once a week with a length of 50 minutes. The German public health care system fully covers psychotherapy fees, but patients must apply for a quota of sessions in predefined steps (12, 24, 60 and 100 sessions). Before applying for psychotherapy sessions, patients are required to take up to seven preparatory and diagnostic sessions. The number of conducted therapy sessions was agreed individually between therapist and patient according to severity and psychotherapeutic focus of the treatment. We included therapies of all lengths to increase external validity. After every fifth session, patients were asked to answer the German 11-item short version of the Hopkins Symptom Checklist (SCL-K11; 42, German Short Version: 43) stated below. On average, patients had M = 57.7 (SD = 29.7, Min = 4, Max = 120) psychotherapy sessions and participated in M = 6.34 (SD = 6.2) repeated assessments for the SCL-K11.

### 2.5 Instruments

#### 2.5.1 Short version of the hopkins symptom checklist

The SCL-K11 assesses the patients experienced symptom severity, including anxiety symptoms (42, German Short Version: 43). Patients are asked to rate the severity of their psychological and somatic symptoms on a five-point-Likert scale from 1 (not at all) to 5 (all the time). The SCL-K11 is reported to have excellent psychometric properties for the 11-item short version (43). Within our total patient sample, internal consistency was high, with a range of Cronbach’s α of.87 -.95 at all measurement occasions.

#### 2.5.2 The operationalised psychodynamic diagnosis structure questionnaire

In the present study, the Operationalised Psychodynamic Diagnosis, second edition (OPD-2) system was used to assess personality functioning (32). The OPD-2 consists of five axes: Axis I= experience of illness and prerequisites for treatment; Axis II= interpersonal relations; Axis III= conflict; Axis IV= structure; Axis V= mental disorders according to ICD-10 (25, 44). Axis IV is also described as *Levels of Structural Integration Axis* (LSIA) (32). From a conceptual perspective, the LSIA exhibits great overlap with the Levels of Personality Functioning Scale (LPFS), according to DSM-5 (25). The OPD-SQ is a self-assessment questionnaire that addresses the basic dimensions of personality structure (Axis IV of OPD), which can also be referred to as personality functioning: perception/cognition, regulation, communication, and attachment (25, 32). It consists of 95 items and comprises eight subscales, with two subscales for each of the aforementioned four dimensions. The subscales are measured by a five-point-Likert scale from ‘0 = Not true at all’ to ‘4 = is completely true’. Higher scores represent greater impairments in personality functioning. Patients answered the OPD-SQ after the clinical intake interview. Within our total patient sample, internal consistency was high for the subscales of perception/cognition of the self (α = .89) and objects (α = .85); regulation of the self (α = .85) and relationships (α = .85); communication with the internal (α = .78) and external (α = .74) world; and attachment to internal (α = .81) and external (α = .77) objects.

#### 2.5.3 Questionnaire for the assessment of adverse and protective childhood experiences

The APC (45) is a self-assessment questionnaire assessing protective childhood experiences (PCE) and adverse childhood experiences (ACE). The questionnaire consists of 59 items (including 17 items for PCE and 40 items for ACE); patients are asked how often they had a specific childhood experience on a five-point-Likert scale from ‘0 = Never’ to ‘4 = Very Often’. The ACE scale comprises questions regarding emotional neglect and abuse, physical neglect and abuse, sexual abuse, traumatic experiences, separation experiences, dysfunctional family situation and missing or dysfunctional peer-group experiences, for example: “In my childhood and youth I was unwanted, I was rejected or made to feel that I was better off not being born” (Item 11). The PCE scale addresses experiences of feeling protected and secure in the family, of respectful interaction and mutual support within the family, of being able to rely on caregivers, of being comforted when feeling sad, and of being accepted and loved for who one was, for example: “In my childhood and youth, I was comforted when I was sad” (Item 23). The APC demonstrates high convergent validity with significant correlations to the childhood trauma questionnaire (CTQ) (45). In addition, Ehrenthal et al. (2020) found a significant negative correlation between PCE, measured by the APC, and the CTQ sum score (45). For this study, the PCE scale (Cronbach’s α = .95) and the ACE scale (Cronbach’s α = .93) were both used (45). We measured the childhood experiences directly after the initial interview.

### 2.6 Pre-power analysis

For our power analysis, we used the R package ‘semtools’ (Version 5.6) (46). Model A assumes every possible effect and will always fit perfectly (47). Therefore, the power analysis was based on Model B, which only assumed indirect effects of childhood experiences on the speed of recovery. Thus, model B had df = 2. Following Kline’s (47) recommendation, we calculated the required sample size for a poor fit test (H_0_: RMSEA =0.1, H_A_: RMSEA = 0.01). With our sample size of N_Anxiety_=312 patients, we achieved a power of.66.

### 2.7 Missing data analysis and multiple imputation

Missing data analysis and imputation was done within the R environment (48) using the package ‘mice’ (Version 3.16) (49). Our subsample of anxiety patients with completed psychotherapies had 13.2% missing data, which resulted in the need of a missing at random assumption and the need of multiple imputation (50). For our imputation model, we included all sociodemographic and psychometric data available and excluded variables with an influx above 0.50 (50). For imputation, we used predictive mean matching. Sum scores were imputed using passive imputation. On average, a variable was predicted with 28.7 predictors (50). The imputation results were checked for plausibility using density, box-and-whisker, and scatter plots (50). For more information, we refer to our online repository, in which the complete analysis is uploaded: https://doi.org/10.11588/data/AJKTKU.

### 2.8 Data analysis strategy

The sample description was done using Microsoft Excel 2019. All further analyses were conducted using R, Version 4.3.1 (48). Calculations were done with the subsample of anxiety patients which completed their psychotherapy. However, for calculation of internal consistencies, we used the total sample of 1646 psychotherapies, also including therapies that treated depression, eating disorders etc., to achieve a more precise estimation of internal consistencies. Imputation was done after calculation of the speed of recovery because imputation of longitudinal data is not robust (50, 51). For more information, we refer to our online repository, in which the complete analysis and results are uploaded: https://doi.org/10.11588/data/AJKTKU.

#### 2.8.1 Calculation of the speed of recovery

All calculations of the speed of recovery were done using the R package ‘nlme’ (Version 3.1-162) (52) and ‘r2mlm’ (Version 0.3.3) (53) using Restricted Maximum Likelihood estimation. Because the symptom severity of patients was right-skewed, we logarithmised symptom severity. Scatterplots of logarithmic symptom severity against therapy hours then showed only a linear relationship. Therefore, we compared two hierarchical linear models to calculate the speed of recovery. We used a null model, which assumed no decrease in symptom severity, and a linear model with random intercept and random slopes with time as the only predictor. Model fit was checked by testing the residuals for normal distribution and heteroscedasticity (54, 55). Model comparison was done using R², MAE, RMSE, and a likelihood ratio test based on the Akaike Information Criterion and Bayesian Information Criterion. Adopting the linear hierarchical model, we extracted the individual slope parameters of the patients. As symptom severity decreases during therapy, we inverted the slope parameter to facilitate interpretation. Thus, a higher value represents a higher speed of recovery.

#### 2.8.2 Structural equation model

All calculations for the structural equation models were conducted with the R packages ‘lavaan’ (Version 0.6-15) (56) and ‘semtools’ (Version 0.5-6) (46) using Maximum Likelihood estimation and their default settings. All tested models were recursive and thus identified (47). Skewness was between -1 and 1 for all variables and excess kurtosis between -4 and 4. Thus, our variables could be considered sufficiently normally distributed (47). We computed our models on each imputed dataset and pooled the results using Rubin’s Rule (50). We first calculated Model A, which assumes effects between all variables. To account for sociodemographic and routinely assessed clinical data, we included age, gender, education (operationalized via the highest educational attainment), and the number of ICD-10 diagnoses in addition to adverse and protective childhood experiences, the four dimensions of personality functioning, and the speed of recovery. In Model B, we removed non- significant paths stemming from Model A if there was a theoretical rationale supported by preliminary studies (47). Both models were checked for global fit using the χ²-Test, the global fit indices SRMR, CFI, the RMSEA, and the 90% confidence interval of the RMSEA. Local fit of both models was assessed using the differences between the observed and calculated correlation matrix. According to Kline (47), a good local fit can be assumed if no difference is greater than 0.1. The models were then compared using a χ²-Test. Finally, we calculated the power post-hoc for our Model B.

### 2.9 Transparency and openness

We report how we determined our sample size, all data exclusions, all manipulations, and all measures in the study, and we follow the Journal Article Reporting Standards (JARS; 57, 58). The analysis code is available at https://doi.org/10.11588/data/AJKTKU. The datasets used and analyzed during the present study cannot be shared due to restrictions by the Ethics Committee of the University of Heidelberg. However, data can be requested from the author J. Nowak (jonathan.nowak@med.uni-heidelberg.de) upon reasonable request and with permission of the Ethics Committee of the University of Heidelberg. Data were analysed using R, Version 4.3.1 (48), all packages are stated above. This study’s design and analysis were not pre-registered.

## 3 Results

All results are available at https://doi.org/10.11588/data/AJKTKU.

### 3.1 Sample description and attrition

Between January 2013 and July 2021, a total of 1890 psychotherapies were conducted at the Heidelberg Institute of Psychotherapy. Written informed consent was given for 1646 psychotherapies. This corresponds to a participation rate of 87.1%. Overall, 648 patients completed their psychotherapy, of whom 312 patients were diagnosed with at least one anxiety disorder (see **Table 1**). As demonstrated in **Table 1**, the subsample of patients with anxiety disorders is slightly younger, has a higher proportion of female patients, and, on average, has one more ICD-10 diagnosis than the patients from the total sample of completed psychotherapies. Furthermore, it appears that the anxiety subsample has a minimally higher proportion of comorbid depression, substance use disorders, posttraumatic stress disorder (PTSD), and eating disorders. Moreover, there is a higher comorbidity of personality disorders in the assessed anxiety patients. Of all patients with anxiety disorders, 32.4% were found to have social phobia, 26.9% specific phobia, 26.0% panic disorder, 25.7% agoraphobia, 16.0% generalized anxiety disorder, 9.9% unspecified anxiety disorder, and 1.28% hypochondriasis.

**Table 1:** Description of all Completed Psychotherapies and Subsample of Anxiety Patients.

	Completed Psychotherapies	Anxiety Patients
N	648	312
Percentage	100%	48.1%
Female	63.6%	68.9%
Age (Mean)	35.6	34.0
Age (SD)	13.1	12.0
Age (Range)	18 – 76	18 – 66
Depressive Disorder	77.5%	81.7%
Personality Disorder	21.1%	28.5%
Substance Abuse	13.7%	15.7%
PTSD	5.4%	6.1%
Eating Disorder	13.5%	14.4%
Number of Diagnoses	2.63	3.60

### 3.2 Speed of recovery

**Table 2** shows the mean decrease in symptom severity per measurement time point. At the beginning of therapy, a sharp drop in symptom severity was identified. As therapy progressed, symptom abatement decreased.

**Table 2:** Mean decrease of SCL-K11 score between measurements.

From	To	Mean	SD	N
Pre	T1	-0.28	0.66	117
T1	T5	-0.07	0.55	214
T5	T10	-0.02	0.67	215
T10	T15	-0.07	0.64	205
T15	T20	-0.03	0.59	200
T20	T25	-0.06	0.55	174
T25	T30	-0.03	0.55	162
T30	T35	-0.10	0.48	156
T35	T40	-0.04	0.54	151
T40	T45	0.00	0.56	141
T45	T50	-0.12	0.50	102
T50	T55	-0.01	0.52	99
T55	T60	-0.04	0.56	76
T60	T65	0.00	0.47	76
T65	T70	-0.08	0.54	73
T70	T75	-0.07	0.60	67
T75	T80	0.08	0.68	43
T80	T85	0.05	0.59	40
T85	T90	-0.09	0.60	35
T90	T95	0.00	0.53	31
T95	T100	0.24	0.37	3
Pre	Post	-0.59	0.76	92

Post measurement was conducted after last therapy session. Timepoint differed between patients. Number of measurements differed due to missing values. Mean values were calculated for patients of all treatment lengths.

Due to missing values, the null model used 287 patients with a total of 3014 measurements and estimated the intercept at 0.65 (SE = 0.02, df = 2727, t = 38.02, p <.001). The standard deviation of the intercept and residual were 0.28 and 0.23, respectively. The intercept explained 59.69% of all variance. Diagnostic plots revealed heteroscedasticity and non-normally distributed residuals. Thus, not all effects have been captured in the model (55). RMSE and MAE of the null model were 0.22 and 0.17 respectively.

The results for the linear model can be seen in **Table 3**. There is a significant linear slope of psychotherapy sessions on logarithmized symptom severity. This means that symptom severity decreases exponentially with the number of therapy sessions. The slope varies between each patient with a standard deviation of 0.02; this variation explains about 6.77% of the total variance. In total, the linear model explains about 74.81% of the variance, 15.12% more than the null model. Residuals are not normally distributed and exhibit heteroskedasticity. Therefore, not all effects are captured in this model either (55). RMSE and MAE of the linear model were 0.19 and 0.14 respectively. The log-likelihood ratio test showed a significant difference between the two models (df_null_ = 3, df_linear_ = 6, Likelihood ratio = 484.68, p <.001). Therefore, we accepted the linear model.

**Table 3:** Linear model of the Speed of recovery.

Fixed effects	Value	SE	df	t	p
Intercept	0.76	0.02	2726	43.67	<.001
Session	-0.02	<0.01	2726	-13.18	<.001
Random effects	SD	Correlation			
Intercept	0.27				
Session	0.02	-.10			
Residual	0.20				
Number of Measurements: 3014			Number of Patients: 287		
	Fixed Effect	Slope Variation	Intercept Variation	Unexplained	
Variance	9.80%	6.77%	58.23%	25.19%	

### 3.3 Analysis of imputed variables

**Table 4** shows the differences between the means and standard deviations of the original data with missing values and the average mean and standard deviation of the 20 imputed data sets. **Table 4** also depicts the standard error, calculated using Rubin’s rule (50). Across all variables that were included in the model, the average mean and standard deviation of the imputed data sets are the same as those of the original data. Patients with missing values, on average, most likely do not differ from patients without missing values.

**Table 4:** Descriptive measures of original and imputed data.

Variable	Original Data		Imputed Data			
	Mean	SD	Mean	Mean SE	SD	SD SE
Attachment	2.04	0.58	2.04	0.18	0.58	0.15
Regulation	1.56	0.60	1.56	0.18	0.60	0.16
Perception	1.61	0.61	1.60	0.19	0.61	0.16
Communication	1.69	0.48	1.69	0.17	0.49	0.14
Speed of Recovery	0.00	0.01	> 0.00	0.02	0.01	0.02
Adverse CE	0.9	0.67	0.9	0.2	0.67	0.16
Protective CE	2.38	0.98	2.38	0.24	0.98	0.2
Age	34.03	12.02	34.03	0.84	11.96	0.7
Gender	0.19	0.46	–	–	–	–
Education	3.82	1.26	3.8	0.27	1.26	0.23
Nr. Diagnoses	3.6	1.63	–	–	–	–

A dash (“-”) represents that no imputation took place, as there were no missing values. For Gender: Female = 0.5, Male = -0.5; Education: 1 = Special School, 2= Junior High School, 3 = Senior High School, 4= Technical College Entrance Qualification 5 = A-Level; CE, Childhood Experience.

### 3.4 Structural equation model

#### 3.4.1 Model A

Model A assumes effects between all variables and therefore has perfect global and local fit to the data. As in the previous study by Kindermann et al. (37), protective childhood experiences had no significant effect on the speed of recovery. Therefore, we removed this path for Model B. Furthermore, age, gender, and education also had no significant effect on the speed of recovery. As there have been previous studies also showing that sociodemographic variables, like age and gender, have no effect on psychotherapy outcome (e.g. 21, 59, 60), we decided to remove those paths for Model B. Although the number of diagnoses had no significant effect on the speed of recovery, there was no theoretical rationale to remove this variable; therefore, we decided to keep this path in the model.

#### 3.4.2 Model B

*Global Fit.* Model B showed mixed global fit indices. The χ²-Test was not significant (χ² = 0.827, df = 4, p = .935) and the model could therefore be tentatively accepted. The CFI and TLI were 1.000 and 1.033, respectively. The RMSEA was.000 (90% CI = [.000;.022]). Since the upper interval of the RMSEA is lower than 0.05, the not-close-fit test and the poor-fit test are significant (47). Therefore, the model fits better than a not-close fitting model. The Likelihood ratio test showed no significant difference to Model A (F = 0.207, df_1 = _4, df_2 = _542.433, p = 0.935).

**Figure 2:**
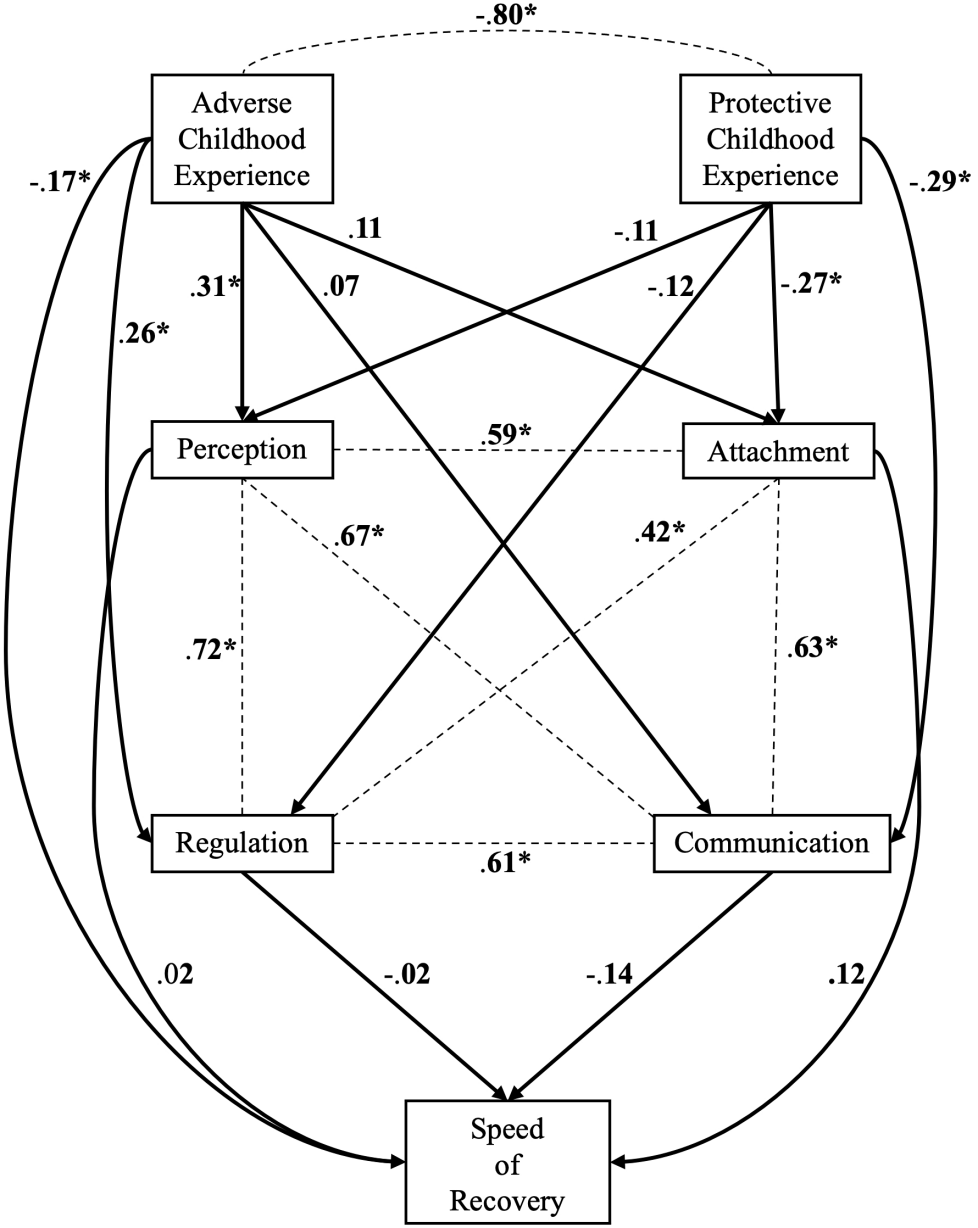
Model B. To achieve readability we omitted all disturbances, variances and all paths connected to the covariates; we only visualized the path corresponding to our hypotheses. The omitted path can be seen in **Table 5**. Dotted Lines represent correlations and arrows represent direct effects. Standardized values are shown. * p <.05.

*Local Fit.* The parameter estimates are shown in **Table 5**. The direct effect of ACEs on the dimensions of perception and regulation within personality functioning were found to be significant. For PCEs, the direct effects on the dimensions of attachment and communication were found to be significant. No direct effects of dimensions of personality functioning on the speed of recovery were significant. A visualization of these paths and their significance can be seen in **Figure 2**. Age and the number of diagnoses both had direct effects on all four dimensions of personality functioning. Gender only had a direct effect on the dimension of attachment; education had no direct effect on any dimension of personality functioning. A comparison of the observed and calculated correlations from Model B showed that no correlation is severely underestimated, as can be seen in **Table 6**. Thus, Model B has a good local fit and can be accepted (47).

**Table 5:** Parameter estimations of the Structural Equation Models.

Regression	Model A				Model B			
	Estimate	SE	p	SV	Estimate	SE	p	SV
Model Parameters								
Attachment ~								
Adverse CE	0.10	0.08	0.20	0.11	0.10	0.08	0.20	0.11
Protective CE	-0.16	0.05	<0.01	-0.27	-0.16	0.05	<0.01	-0.27
Regulation ~								
Adverse CE	0.24	0.08	<0.01	0.26	0.24	0.08	<0.01	0.26
Protective CE	-0.07	0.06	0.20	-0.12	-0.07	0.06	0.20	-0.12
Perception ~								
Adverse CE	0.28	0.08	<0.01	0.31	0.28	0.08	<0.01	0.31
Protective CE	-0.07	0.05	0.22	-0.11	-0.07	0.05	0.22	-0.11
Communication ~								
Adverse CE	0.05	0.07	0.45	0.07	0.05	0.07	0.45	0.07
Protective CE	-0.15	0.04	<0.01	-0.29	-0.15	0.04	0.00	-0.29
Speed of Recovery ~								
Adverse CE	0.00	<0.01	0.03	-0.23	0.00	0.00	0.01	-0.17
Protective CE	0.00	<0.01	0.48	-0.07	–	–	–	–
Attachment	0.00	<0.01	0.23	0.11	0.00	0.00	0.17	0.12
Regulation	0.00	<0.01	0.85	-0.02	0.00	0.00	0.85	-0.02
Communication	0.00	<0.01	0.18	-0.13	0.00	0.00	0.17	-0.14
Perception	0.00	<0.01	0.77	0.03	0.00	0.00	0.86	0.02
Covariates								
Attachment ~								
Age	-0.01	<0.01	0.01	-0.16	-0.01	0.00	0.01	-0.16
Gender	0.20	0.07	<0.01	0.16	0.20	0.07	<0.01	0.16
Education	-0.02	0.03	0.47	-0.04	-0.02	0.03	0.47	-0.04
Nr. Diagnoses	0.07	0.02	<0.01	0.20	0.07	0.02	<0.01	0.20
Regulation ~								
Age	-0.01	<0.01	<0.01	-0.17	-0.01	0.00	<0.01	-0.17
Gender	-0.03	0.07	0.66	-0.02	-0.03	0.07	0.66	-0.02
Education	0.00	0.03	0.93	-0.01	0.00	0.03	0.93	-0.01
Nr. Diagnoses	0.06	0.02	<0.01	0.17	0.06	0.02	<0.01	0.17
Perception ~								
Age	-0.01	0.00	<0.01	-0.20	-0.01	0.00	<0.01	-0.20
Gender	0.11	0.07	0.09	0.09	0.11	0.07	0.09	0.09
Education	0.00	0.03	0.88	-0.01	0.00	0.03	0.88	-0.01
Nr. Diagnoses	0.08	0.02	<0.01	0.22	0.08	0.02	<0.01	0.22
Communication ~								
Age	-0.01	0.00	<0.01	-0.25	-0.01	0.00	<0.01	-0.25
Gender	-0.01	0.06	0.86	-0.01	-0.01	0.06	0.85	-0.01
Education	-0.02	0.02	0.50	-0.04	-0.02	0.02	0.50	-0.04
Nr. Diagnoses	0.06	0.02	<0.01	0.19	0.06	0.02	<0.01	0.19
Speed of Recovery ~								
Age	0.00	0.00	0.55	0.04	–	–	–	–
Gender	0.00	0.00	0.84	0.01	–	–	–	–
Education	0.00	0.00	0.99	0.00	–	–	–	–
Nr. Diagnoses	0.00	0.00	0.85	0.01	0.00	0.00	0.89	0.01
Covariance	Estimate	SE	p	SV	Estimate	SE	p	SV
Model Parameters								
Adverse CE ~~								
Protective CE	-0.53	0.05	<0.01	-0.80	-0.53	0.05	<0.01	-0.80
Attachment ~~								
Regulation	0.11	0.02	<0.01	0.42	0.11	0.02	<0.01	0.42
Communication	0.13	0.02	<0.01	0.63	0.13	0.02	<0.01	0.63
Perception	0.15	0.02	<0.01	0.59	0.15	0.02	<0.01	0.59
Regulation ~~								
Communication	0.14	0.02	<0.01	0.61	0.14	0.02	<0.01	0.61
Perception	0.20	0.02	<0.01	0.72	0.20	0.02	<0.01	0.72
Perception ~~								
Communication	0.15	0.02	<0.01	0.67	0.15	0.02	<0.01	0.67
Covariates								
Age ~~								
Education	-4.94	0.94	<0.01	-0.33	-4.94	0.94	0.00	-0.33
Nr. Diagnoses	-1.62	1.16	0.16	-0.08	-1.62	1.16	0.16	-0.08
Gender	0.15	0.33	0.65	0.03	0.15	0.33	0.65	0.03
Gender ~~								
Education	0.03	0.04	0.38	0.05	0.03	0.04	0.38	0.05
Nr. Diagnoses	0.08	0.05	0.07	0.11	0.08	0.05	0.07	0.11
Education ~~								
Nr. Diagnoses	-0.31	0.12	0.01	-0.15	-0.31	0.12	0.01	-0.15
Adverse CE ~~								
Gender	0.04	0.02	0.04	0.12	0.04	0.02	0.04	0.12
Age	0.84	0.48	0.08	0.11	0.84	0.48	0.08	0.11
Education	-0.24	0.05	<0.01	-0.28	-0.24	0.05	<0.01	-0.28
Nr. Diagnoses	0.31	0.07	<0.01	0.29	0.31	0.07	<0.01	0.29
Protective CE ~~								
Gender	-0.02	0.03	0.39	-0.05	-0.02	0.03	0.39	-0.05
Age	-2.08	0.71	<0.01	-0.18	-2.08	0.71	<0.01	-0.18
Education	0.36	0.08	<0.01	0.30	0.36	0.08	<0.01	0.30
Nr. Diagnoses	-0.36	0.10	<0.01	-0.22	-0.36	0.10	<0.01	-0.22
Variance	Estimate	SE	p	SV	Estimate	SE	p	SV
Model Parameters								
Attachment	0.25	0.02	<0.01	0.73	0.25	0.02	<0.01	0.73
Regulation	0.28	0.02	<0.01	0.79	0.28	0.02	<0.01	0.79
Perception	0.26	0.02	<0.01	0.70	0.26	0.02	<0.01	0.70
Communication	0.18	0.02	<0.01	0.77	0.18	0.02	<0.01	0.77
Speed of Recovery	0.00	0.00	<0.01	0.96	0.00	0.00	<0.01	0.96
Adverse CE	0.45	0.04	<0.01	1.00	0.45	0.04	<0.01	1.00
Protective CE	0.95	0.08	<0.01	1.00	0.95	0.08	<0.01	1.00
Covariates								
Age	142.56	12.02	<0.01	1.00	142.56	11.99	<0.01	1.00
Gender	0.21	0.02	<0.01	1.00	0.21	0.02	<0.01	1.00
Education	1.58	0.13	<0.01	1.00	1.58	0.13	<0.01	1.00
Nr. Diagnoses	2.65	0.22	<0.01	1.00	2.65	0.22	<0.01	1.00
R-Square	Estimate				Estimate			
Model Parameters								
Attachment	0.27				0.27			
Regulation	0.21				0.21			
Perception	0.30				0.30			
Communication	0.23				0.23			
Speed of Recovery	0.04				0.04			
Adverse CE	0.00				0.00			
Protective CE	0.00				0.00			
Covariates								
Age	0.00				0.00			
Gender	0.00				0.00			
Education	0.00				0.00			
Nr. Diagnoses	0.00				0.00			

S.V, Standardized Value. CE, Childhood Experience. “~” = Variable is regressed by (…). “~~” = Variable correlates with (…).

**Table 6:** Differences between Correlation Matrix of Model B and observed Correlations.

	1	2	3	4	5	6	7	8	9	10	11
1. Attachment	0										
2. Regulation	0	0									
3. Perception	0	0	0								
4. Communication	0	0	0	0							
5. Speed of Recovery	0	0	0	0	0						
6. Adverse CE	0	0	0	0	0	0					
7. Protective CE	0	0	0	0	-0.03	0	0				
8. Age	0	0	0	0	0.04	0	0	0			
9. Gender	0	0	0	0	0.01	0	0	0	0		
10. Education	0	0	0	0	-0.01	0	0	0	0	0	
11. Nr. Diagnoses	0	0	0	0	0	0	0	0	0	0	0

Correlations represent Bollen correlations. CE, Childhood Experience.

### 3.5 Post-hoc-power analysis

For our post-hoc power analysis, we calculated the power of the poor-fit test assuming H_0_ = .10 and H_A_ = .01 with df = 4 and n = 312 using the ‘semtools’ package (46). We obtained a power of .77.

### 3.6 Explained variances

According to Kline (47), R² equals 1- Disturbance (= error + unknown causes) for endogenous variables. Accordingly, we obtained a R² of 1 - 0.964 = .036 for the speed of recovery. Therefore, our model explains 3.6% of variance of speed of recovery for patients with anxiety disorders.

## 4 Discussion

The present study examined adverse and protective childhood experiences as well as personality functioning as potential predictors for the speed of recovery in psychotherapy of patients with anxiety disorders. A higher number of ACEs was found to predict a lower speed of recovery during psychotherapy in terms of a direct effect. The effect of ACEs on the speed of recovery was still present after controlling for age, gender, education, and number of diagnoses. Furthermore, a higher number of ACEs was associated with greater impairments in the abilities of perception and regulation as dimensions of personality functioning. However, none of the dimensions in personality functioning showed significant associations with the speed of recovery in psychotherapy. Therefore, concerning the variables included in our model, ACEs seem to have direct but no indirect effects on the speed of recovery in anxiety disorders. On the other hand, a higher number of PCEs was associated with fewer impairments in the abilities of communication and attachment as dimensions of personality functioning, but had no direct effect on the speed of recovery during psychotherapy.

Our main result was the finding that ACEs are a predictor of a lower speed of recovery during psychotherapy in patients with anxiety disorders. There are few previous studies that have examined predictors of treatment outcome across different anxiety disorders. In 2016, Jakubovski & Bloch were able to identify comorbid depression and a low socioeconomic status as the most important predictors of poor therapy outcome across different anxiety disorders (61). In contrast, an association between a good treatment outcome on the one hand and positive treatment expectancy and high self-efficacy expectancy on the other hand could be demonstrated (61). In a 2012 study, Wolitzky-Taylor et al. (2012) analyzed non-specific predictors of treatment outcome for different anxiety disorders in comparison of cognitive behavioral therapy (CBT) to acceptance and commitment therapy (ACT) (62). It was shown that higher baseline neuroticism was associated with a poorer outcome across the two different treatment conditions; ethnicity, age, gender, and baseline severity of anxiety disorder were not predictive of treatment outcome (62). In the present study, we found a direct effect of ACEs on the speed of recovery during psychotherapy in patients with anxiety disorders, which persisted after controlling for age, gender, education, and number of diagnoses. In previous studies, the effects of ACEs on treatment outcome have been studied primarily in relation to depressive disorders, wherein a higher number of ACEs was associated with a higher symptom severity and complexity, as well as with a poorer treatment outcome in patients with depressive disorder, assuming a dose-response relationship of experienced ACEs (12, 31, 63, 64). A similar relationship between ACEs and treatment outcome has been reported for borderline personality disorder (65), substance abuse disorders (66) as well as for a heterogenous sample of psychiatric outpatients (10). Previous studies explained the association between ACEs and poorer treatment outcome in different ways: On the one hand, ACEs are assumed to lead to a higher psychopathological symptom severity and to a greater number of comorbidities, which in turn could be associated with a worse therapy outcome (67, 68). On the other hand, previous studies indicated that individuals with ACEs exhibit a high risk of developing insecure attachment, which may lead to disturbances in therapeutic alliance in psychotherapeutic treatment, ultimately resulting in poorer outcome (68–73). Overall, our finding that a history of ACEs may lead to a lower speed of recovery in psychotherapy is consistent with the results of previous studies on other mental disorders.

In our study, childhood experiences were assessed retrospectively, namely once before the beginning of psychotherapy. However, with regard to childhood experiences, some studies found evidence that the recollection of these memories might change during psychotherapy (74). In a recent study, Ernst et al. (2023) assessed self-reported childhood adversity before and after 7-8 weeks of inpatient psychotherapy (75). After treatment, patients were shown to report some kinds of abuse and neglect as more severe (75). Furthermore, these changes in the recollection were found to be related to a reduction of depressive symptoms, indicating that a new evaluation of past events might be connected to symptom improvement (75). However, other studies investigating the stability of self-reported childhood adversity over time found no significant changes (76, 77). In their 2018 study, Frampton et al. (2018) examined the effects of depression on the recollection of ACEs and found that changes in depressive symptoms did not correspond with changes in the recollection of ACEs (77). Frampton et al. (2018) concluded that ACE measurements seem to be reliable and stable over time, regardless of depression status (77). Overall, there are heterogeneous findings regarding the stability of self-reported ACEs over time and their possible impact on the course of treatment. Therefore, in order to further investigate how potential changes in recollection might affect recovery during psychotherapy and to what extent psychopathology or other factors might influence the recollection of childhood adversity, future research should assess the recollection of childhood experiences pre- and post-treatment with regard to different diagnostic entities.

With regard to the relationship between specific personality characteristics and anxiety disorders, previous studies have mainly focused on personality disorder traits (78). Epidemiological studies have demonstrated a high comorbidity with personality disorders of Cluster C in anxiety patients, with the avoidant personality disorder occurring most frequently, followed by the obsessive-compulsive, and the dependent personality disorder (19). In a 2014 study, Skodol et al. found that a variety of personality disorders predicted the persistence of anxiety disorders over 3 years (79). Therefore, personality psychopathology seems to be not only a risk factor but also a maintaining factor for anxiety disorders. Skodol et al. concluded their study with the recommendation that personality psychopathology should be assessed and addressed in psychotherapeutic treatment for all patients with anxiety disorders (79). In their 2018 study, Doering et al. (2018) investigated personality functioning in patients with generalized anxiety disorder, panic disorder, and phobia. Significant impairments of personality functioning were found in all three patient groups compared to a control group (21). However, they found no differences in personality functioning between patients with different anxiety disorders. In a recent cross-sectional study, Nowak et al. (2023) examined personality functioning according to OPD in anxiety disorders, comparing patients with generalized anxiety disorder, panic disorder and phobic disorders with non-anxiety patients (26). It was discovered that anxiety patients showed greater impairments in the overall mean as well as in most dimensions of personality functioning compared to patients without anxiety disorder. More specifically, in comparison to other anxiety disorders, patients with phobic disorders showed significantly greater impairments in the ability to communicate with the external world as one dimension of personality functioning (26). Furthermore, evidence was found that patients with generalized anxiety disorder have greater impairments in the ability of self-regulation than patients with other anxiety disorders (26). In summary, our results underscore the findings from previous studies that anxiety disorders are associated with impairments in personality functioning.

The present study provided further evidence that specific impairments in personality functioning in patients with anxiety disorders seem to be associated with ACEs. However, we found no direct effects of personality functioning on the speed of recovery during psychotherapy. Our findings are partially consistent with the recent study by Kerber et al. (2023), in which ACEs and personality functioning were examined as predictors of anxiety symptoms, depressiveness, and somatization (36). In this cross-sectional study, while it was shown that ACEs were positively associated with psychopathology, an association between ACEs and impairments of personality functioning was also found. Furthermore, in a recent study, Dagnino et al. (2020) investigated the relationship between ACEs, personality functioning, and depressive symptomatology and found that personality functioning mediated the relationship between ACEs and depressive symptoms; thus, this finding also implies indirect effects of ACEs on depressive symptoms via personality functioning (31). However, most of the previous studies did not clearly differentiate which individual dimensions of personality functioning may have an effect on symptom severity or on the speed of recovery in psychotherapy. Kindermann et al. (2023) recently found that ACEs had direct as well as indirect effects on the speed of recovery by being associated with impairments in the communication dimension of personality functioning, which in turn was associated with a lower speed of recovery (37). In contrast, the present study found direct, but no indirect, effects of ACEs on the speed of recovery in patients with anxiety disorders. Therefore, while our hypotheses (a) and (b) were confirmed, hypothesis (c) could not be confirmed. A possible explanation for this finding could be that the effect of personality functioning on the speed of recovery during psychotherapy is rather small and emerges as non-significant due to the relatively small group size of the present study. This assumption may be underscored by the previous study by Kindermann et al. (2023), in which the effect sizes of personality functioning on the speed of recovery were also found to be small (37). Furthermore, recent studies assumed that, in particular, *changes* in personality functioning during psychotherapy may be predictive of treatment outcome (27, 30). This notion could indicate that, although the present study found that the *pre-treatment* assessed personality functioning was not predictive for the speed of recovery, *changes* in personality functioning during psychotherapy may be predictive. Therefore, the improvement of personality functioning in anxiety patients should remain an important target of psychotherapy.

In previous studies, ACEs were mainly related to a higher risk of developing mental illness, higher symptom burden, and poorer treatment outcome (10–12). However, childhood adversity was found to be not necessarily associated with psychosocial distress (80). With regard to traumatic experiences, it was shown that stressful life events may also lead to positive psychological changes, which can be referred to as posttraumatic growth (PTG; 81). The concept of PTG refers to subjectively experienced positive changes, such as an increased appreciation for one’s life, increased experience of closeness in relationships, or an increased recognition of personal strengths in the aftermath of traumatic events (81). Previous studies were able to identify PTG in individuals having experienced sexual abuse (82) or neglect (83) in their childhood. In a 2021 study, Tranter et al. (2021) investigated the association between ACEs and PTG and identified emotional resilience as a mediating factor, which could represent an important target in psychotherapeutic treatment of individuals that have experienced childhood adversity (84). Furthermore, Tranter et al. (2021) concluded that psychotherapeutic interventions could focus on enhancing the awareness of potential positive changes after childhood adversity to encourage the patients to recognize possible gains from their childhood experiences (84). However, it still remains unclear which other variables may influence whether ACEs result in psychological distress or psychological growth.

Overall, the results of the present study emphasized the important role that childhood adversity plays in the course of psychotherapy for patients with anxiety disorders. Consequently, ACEs should be routinely assessed before starting psychotherapy and thoroughly addressed during psychotherapy in patients with anxiety disorders. Further research is needed to specify the complex relationships between childhood experiences, individual dimensions of personality functioning, and their possible effect on the course and outcome of psychotherapy in anxiety disorders.

## 5 Limitations

The present study was an exploratory study designed to collect first data on the relationship between ACEs, personality functioning, and the speed of recovery during psychotherapy in patients with anxiety disorders. Several limitations of the presented study should be addressed, with some related to the exploratory approach of the study. First of all, it must be pointed out that we assessed childhood experiences and dimensions of personality functioning simultaneously. Although theoretical considerations and the results of previous studies may lead to the conclusion that childhood experiences could influence the dimensions of personality functioning, the simultaneous assessment of both variables ultimately does not allow any statement on causality. One could possibly argue that impairments in specific dimensions of personality functioning may lead to distorted childhood memories, which in turn lead to a lower speed of recovery in psychotherapy. Another limitation is the fact that the present study only assessed childhood experiences pre-treatment; however, previous studies could demonstrate that the recollection of adverse childhood experiences can change during psychotherapy and can therefore be considered as not being perfectly stable over time (75). Furthermore, a major limitation is the fact that the present study only assessed personality functioning pre-treatment and did not assess changes in personality functioning during therapy, which in turn could interact with the speed of recovery. Future studies should therefore also investigate changes in the recollection of childhood memories and/or changes in personality functioning in parallel with the speed of recovery during psychotherapy. The patients’ heterogeneity with regard to the different anxiety disorders (i.e. panic disorder, generalized anxiety disorder, phobic disorders) may be a further limitation of our results, as individual and diagnosis-specific aspects of personality functioning and the speed of recovery were not addressed accordingly. Thus, it cannot be excluded that the relationship between ACEs, PCEs, and personality functioning differs across diagnostic entities.

## 6 Conclusion

The presence of ACEs had a direct effect on the speed of recovery during psychodynamic treatment of patients with anxiety disorders. Moreover, ACEs and PCEs were shown to be associated with specific dimensions of personality functioning. Therefore, evaluating childhood experiences before starting psychotherapy could be a relevant component for treatment planning. Future studies should focus particularly on the complex relationship between childhood experiences, personality functioning, and treatment outcome in different mental disorders.

## Data availability statement

The data that support the findings of this study were collected at the outpatient training clinic for psychodynamic therapy at the University Hospital Heidelberg, Germany (https://www.klinikum.uni-heidelberg.de/zentrum-fuer-psychosoziale-medizin-zpm/hip/heidelberger-institut-fuer-psychotherapie-hip). Data can be supplied by the author J. Nowak (jonathan.nowak@med.uni-heidelberg.de) upon reasonable request and after permission has been granted by the Ethics Committee of the University of Heidelberg. The analysis code of our data analysis is publicly available at: https://doi.org/10.11588/data/AJKTKU.

## Ethics statement

The studies involving humans were approved by Ethics Committee of the Medical Faculty of the Heidelberg University. The studies were conducted in accordance with the local legislation and institutional requirements. The participants provided their written informed consent to participate in this study.

## Author contributions

JN: Methodology, Writing – review & editing, Writing – original draft, Conceptualization. CN: Methodology, Writing – review & editing, Writing – original draft, Supervision, Project administration, Conceptualization. IR: Writing – review & editing, Writing – original draft, Visualization, Software, Methodology, Formal analysis, Conceptualization. MO: Writing – review & editing, Software, Formal analysis, Data curation. HF: Writing – review & editing, Supervision. DK: Writing – review & editing, Writing – original draft, Supervision, Methodology, Conceptualization.
